# Chest roentgenography is complementary to interferon-gamma release assay in latent tuberculosis infection screening of rheumatic patients

**DOI:** 10.1186/s12890-020-01274-9

**Published:** 2020-08-31

**Authors:** Ping-Huai Wang, Chou-Han Lin, Ting-Hui Chang, Chien-Sheng Wu

**Affiliations:** 1 Division of Thoracic Medicine, Department of Internal Medicine, Far Eastern Memorial Hospital, New Taipei City, Taiwan; 2 Oriental Institute of Technology, New Taipei City, Taiwan; 3 Division of Rheumatology, Department of Internal Medicine, Far Eastern Memorial Hospital, No. 21 Sec 2 Nan-Ya South Road, Distinct Banchiao, 220 New Taipei City, Taiwan

**Keywords:** Latent tuberculosis, Interferon-gamma release assay, Chest roentgenography, Biologics

## Abstract

**Background:**

A study of latent tuberculosis infection (LTBI) burden by chest roentgenography (CXR) with reference to interferon-gamma release assay (IGRA) is still lacking in rheumatic patients of an intermediate tuberculosis burden area.

**Methods:**

We retrospectively reviewed clinical data of patients with rheumatoid arthritis (RA), ankylosing spondylitis (AS), or psoriatic arthritis (PsA) receiving LTBI screening for biologics from Jan 2013 to April 2014.

**Results:**

A total of 238 rheumatic patients who underwent LTBI screening were included in this study, of whom 46 (19.3%) had positive IGRA tests, 178 (74.8%) had negative results, and 14 (5.9%) had indeterminate results. Radiological findings suggesting healed tuberculosis (CXR-old-TB) were found in 18.1% of all patients, 23.9% in the IGRA -positive patients vs 16.9% in the IGRA-negative patients (OR 1.55 95% CI: 0.71–3.39, *p* = 0.27). Forty (40/46, 87.0%) IGRA-positive patients received isoniazid prophylaxis and 77.5% of them finished treatment. Six patients developed adverse effects of isoniazid treatment, resulting in an overall number needed to harm (NNH) of 6.7 (40/6). IGRA-non-positive patients with old TB-suggestive CXR comprised 13.4% (32/238) of all our rheumatic patients, and one of them developed pulmonary tuberculosis within one year after screening.

**Conclusions:**

LTBI disease burden in rheumatic patients is substantial according to the estimation of CXR and IGRA screening. Correlation between CXR and IGRA is not significant in rheumatic patients, which implies their complementary roles. IGRA-non-positive patients with old TB-suggestive CXR comprise a significant portion in rheumatic patients and merit cautious follow-up by rheumatologists, tuberculosis specialists, and pulmonologists.

## Background

Tumor necrosis factor-alpha (TNF-α) inhibitors have revolutionized the treatment of rheumatic diseases including rheumatoid arthritis (RA), ankylosing spondylitis (AS), and psoriatic arthritis (PsA). However, TNF-α plays a critical role in immune reactions against *Mycobacterium tuberculosis* (MTB) [1, 2], and interference of TNF-α by biologic agents may affect anti-MTB immunity, potentially leading to reactivation of latent MTB [3, 4]. Previous studies showed that anti-TNF therapy was associated with an increased risk of MTB activation [5, 6]. Latent tuberculosis infection (LTBI) not only complicates the treatment course but also threatens public health.

To date, there is no gold standard for the detection of LTBI. Present methods for screening latent tuberculosis include chest roentgenography (CXR), tuberculin skin test (TST), and interferon-gamma release assays (IGRAs). IGRAs have been developed to measure the production of interferon-gamma in lymphocytes upon stimulation with unique MTB antigens. Such IGRAs have been shown to have minimal cross-reaction with non-tuberculous mycobacteria (NTM). The positive and negative predictive values of IGRAs for developing active tuberculosis is higher than those of the TST in general populations [7]. The specificity of IGRAs has also been reported to be superior to the TST in Bacillus Calmette-Guerin (BCG)-vaccinated populations [8, 9] and immunocompromised patients [10]. Therefore, IGRAs is widely used to replace the TST as a screening tool for LTBI in rheumatic patients [11], especially in areas with BCG vaccination programs [12], such as universal vaccination policy in Taiwan.

LTBI screening and prophylaxis in patients with rheumatic diseases are critical issues in Asia [13]. The incidence of MTB in the general population in Taiwan between 2013 and 2015 was around 45–49 per 100,000 person-years or about 0.05 per 100 patient-years [14]. However, the incidence rates of tuberculosis in RA patients treated with adalimumab and etanercept between 2006 and 2011 were 1.41–1.62 and 0.57–0.68 events per 100 person-years, respectively [15, 16]. LTBI prophylaxis in rheumatic patients achieved a various degree of success in different countries [17–19]. In Taiwan, TNF inhibitor (TNFi) was reimbursed by National Health Insurance since 2003, and LTBI screening and prophylaxis policy of rheumatic patients with biologic therapy was announced by Taiwan Food and Drug Administration in 2012, after which the incidence of tuberculosis tended to decrease but did not reach statistical significance [20]. Therefore, there is an unmet need in LTBI screening.

In addition to IGRAs, CXR is included in the Taiwan LTBI risk management plan of biologics [21]. Current guidelines regarding the use of chest radiography in screening LTBI have mixed recommendations. Some suggest a chest radiograph after a positive IGRA [22]. On the contrary, others suggest chest radiography regardless of the result of interferon-gamma release assay (IGRA) or tuberculin skin test (TST) [23]. A universally accepted standard of care in this issue has not yet established. Furthermore, the studies on the correlation between CXR and IGRA reveal different results in different population, such as TB-exposed or health care workers [24, 25], and studies on rheumatic patients are limited. In addition, the burden of LTBI estimated by CXR besides IGRA is unclear in areas of intermediate MTB risk, though the healed but untreated fibrotic lesions in CXR are associated with a risk for progression to active TB [26]. Therefore, we conducted this retrospective study to investigate the CXR findings in the LTBI screening. We also investigated CXR and IGRA correlations and reported the results of isoniazid prophylaxis.

## Patients and methods

Patients with RA, AS, or PsA, who received their first LTBI screening as candidates of biologics or current users between January 2013 and April 2014 in a tertiary-care hospital, were included in this retrospective study. All patients received a CXR with tuberculosis IGRA test (QuantiFERON-TB Gold In-Tube, Cellestis, Australia). This retrospective study was approved by the Institutional Review Board of Far Eastern Memorial Hospital (IRB-105066-E). The medical records of all patients were reviewed until one year after the screening, and a history of previous MTB infection and close contact with patients with MTB were also identified.

According to the Taiwan Rheumatology Association Recommendations, candidates for biologic therapy should receive initial IGRA and CXR screening, and current users should receive yearly IGRA screening and CXR every six months [21]. Nine months of daily isoniazid 300 mg therapy (9H) is recommended for LTBI chemoprevention. Patients with LTBI are defined as those with positive IGRA but without CXR or other evidence of active MTB. If IGRA tests are negative or indeterminate, LTBI diagnosis depends on clinical conditions and CXR findings. If the patients with LTBI are unwilling to take or intolerant to isoniazid, they can receive CXR follow-up every three months by tuberculosis specialist or pulmonologists.

Two pulmonologists reviewed the CXR taken within three months of the IGRA screening. Apical pleural thickening, interstitial granuloma calcification, thin-wall cysts, pericardial calcifications, fibrotic lesions seen as irregular linear opacities, and localized bronchiectasis were classified as old MTB infection-suggesting lesions [27]. The calcification of mediastinal or hilar lymph nodes without a history of exposure to dust was also considered as possible healed old MTB lesions [27]. The patients presented with the above-mentioned radiologic features were defined as CXR-old-TB group, and the CXR-non-TB group was defined as those without old pulmonary MTB-suggestive lesions. The examinations and interpretation of IGRA tests complied with the recommendations of the manufacturer (QuantiFERON-TB Gold In-Tube, Cellestis, Australia). Hepatitis was defined as alanine aminotransferase (ALT) more than 100 IU/L.

All continuous data were expressed as mean ± SD, and categorical data were expressed as percentage unless otherwise stated. Statistical analysis was performed using SPSS software version 18 (SPSS Inc., Chicago, IL, USA). Continuous data were compared by the Student’s t-test or ANOVA, and categorical data were compared by the Chi-square test. Statistical significance was defined as *p* < 0.05.

## Results

### Clinical characteristics of the patients receiving LTBI screening

A total of 238 patients were reviewed, including 183 (76.9%) with RA, 34 (14.3%) with AS, and 21 (8.8%) with PsA (Table 1). The patients with RA were female predominant and significantly older than those with AS and PsA (*p* < 0.001 by ANOVA). The anti-rheumatic drugs were listed in Table 1. Since biologics were reimbursed since 2003 before the 2012 LTBI screening policy in Taiwan, many patients had received biologics before their first IGRA screening. In the patients with RA, AS, and PsA, the IGRA positive rates were 20.2, 23.5, and 4.8%, and the old TB-suggestive CXR presented in 18.6, 17.6, and 14.3%, respectively. We also analysed the CXR and IGRA screening results according to patients with or without prior biologic use, which revealed no statistically significant difference between the two groups (Table 2).

**Table 1:** Demographic data, latent tuberculosis screening results and medication of patients with rheumatic diseases

	Total *N* = 238	RA *N* = 183	AS *N* = 34	PsA *N* = 21
Age (mean ± SD)	55.4 ± 14.8	56.9 ± 13.2^a^	41.8 ± 14.7	41.5 ± 13.0
Sex (M/F)	73/165	34/149 ^b^	27/7	12/9
**Medication** n (%)				
MTX	104 (43.7)	102 (55.7)	1 (2.9)	1 (4.8)
LEF	78 (33.8)	62 (33.9)	1 (2.9)	15 (71.4)
SSZ	128 (53.8)	92 (50.3)	26 (76.5)	10 (47.6)
HCQ	61 (25.6)	59 (32.2)	1 (2.9)	1 (4.8)
Glucocorticoid	127 (53.4)	114 (62.3)	4 (11.8)	9 (42.9)
Pred. dose (mg)	3.6	4.2	0.7	3.0
NSAIDs	194 (81.5)	150 (82.0)	30 (88.2)	14 (66.7)
Biologics	207 (87.0)	155 (84.7)	32 (94.1)	20 (95.2)
TNFi	179 (75.2)	131 (71.6)	32 (94.1)	16 (76.2)
non-TNFi	28 (11.8)	24 (13.1)	0 (0)	4 (19.0)
**Screening results**				
IGRA P/N/IND n (%)	46/178/14 (19.3/74.8/5.9)	37/132/14 (20.2/72.1/7.7)	8/26/0 (23.5/76.5/0)	1/20/0 (4.8/95.2/0)
CXR-old-TB n (%)	43 (18.1)	34 (18.6)	6 (17.6)	3 (14.3)

^a^ RA vs. AS or PsA (*p* < 0.001 by ANOVA). ^b^ RA vs AS or PsA (*p* < 0.001by Chi-square test)

*RA* rheumatoid arthritis, *AS* ankylosing spondylosis, *PsA* psoriatic arthritis, *MTX* methotrexate, *LEF* leflunomide, *Pre. dose* glucocorticoid equivalent dose in prednisolone, *SSZ* sulfasalazine, *HCQ* hydroxychloroquine, *NSAID* non-steroidal anti-inflammatory drug, *TNFi* TNF- inhibitor, *IGRA* interferon-gamma release assay, *IND* indeterminate, *N* negative, *P* positive, *CXR-old-TB* radiological evidence of previous tuberculosis infection

**Table 2:** The comparison of the IGRA and chest radiographic findings in biologic naïve and biologic-treated rheumatic patients

	Biologic naïve *N* = 31	Biologic user *N* = 207	*p*
Age	57.5 ± 16.1	52.8 ± 14.6	0.10 ^a^
Sex (M:F)	10/21	63/144	0.84 ^b^
RA/AS/PsA	28/2/1	155/32/20	0.16 ^b^
IGRA findings (Pos/Neg/IND)	10/19/2	36/159/12	0.14 ^b^
CXR-old-TB n (%)	4 (12.9)	39 (18.8)	0.42 ^b^

^a^ Student’s t test. ^b^ Chi-square test

*RA* rheumatoid arthritis, *AS* ankylosing spondylitis, *PsA* psoriatic arthritis, *IGRA* interferon gamma release assay, *Pos* positive, *Neg* negative, *IND* indeterminate

### Correlations between chest roentgenography and IGRA screening results

As shown in Tables 3, 46 (19.3%) of all patients had positive IGRA test results, 14 (5.9%) had indeterminate results, and 178 (74.8%) had negative results. The IGRA-negative group was younger than the other two groups (*p* < 0.01 by ANOVA test). CXR suggestive of tuberculosis sequelae were noted in 18,1% of patients. In the IGRA-positive group, 11 (23.9%) patients had radiological findings suggesting tuberculosis sequelae; two of them completed pulmonary tuberculosis treatment 21 and 29 years before the IGRA screening. Only 30 (16.9%) of the IGRA-negative group had TB-suggestive lesions. The prevalence of old-TB suggestive findings was not significantly higher in the IGRA-positive group than in the IGRA-negative group (OR 1.55, 95% CI: 0.71–3.39, *p* = 0.27). Among the patients with positive IGRA results, the IGRA values were not significantly different between the CXR-old-TB and CXR-non-TB groups (2.35 ± 2.43 vs. 1.91 ± 1.74 IU/ml, respectively, *p* = 0.51). In 192 patients with negative or indeterminate IGRA tests, 32 patients had radiological findings suggesting healed MTB. Fibrotic lesions presented in nineteen of them (59.4%, 19/32) (Table 3); and one patient with an indeterminate IGRA result completed MTB treatment 21 years before the screening.

**Table 3:** The association between IGRA results and chest radiologic findings of healed tuberculous infections

IGRA	Positive *N* = 46	Negative *N* = 178	Indeterminate *N* = 14
Age	57.9 ± 11.2	51.7 ± 15.6 ^a^	59.2 ± 11.1
Sex (M/F)	19/27	52/126	2/12
CXR-old-TB n (%)	11 (23.9)^b,c^	30 (16.9) ^d^	2 (14.3) ^e^
Fibrotic lesions	7^c^	17	2
Calcified nodules	3	10	0
Local bronchiectasis	1	6	0
Thin wall cysts	1	0	0
Hilar calcifications	0	0	0

^a^ *p* < 0.01 by ANOVA

^b^ Positive vs negative: odd ratio 1.55, 95% CI: 0.71–3.39, *p* = 0.27

^c^ One subject had *Mycobacterium gordonae* colonization; Two subjects had previous history of complete pulmonary tuberculosis treatment

^d^ One subject had *Mycobacterium tuberculosis* infection during follow-up period

^e^ One subject had previous history of complete pulmonary tuberculosis treatment

*IGRA* interferon gamma release assay, *CXR-old-TB* radiographic findings suggestive of pulmonary tuberculosis sequelae

### Isoniazid prophylaxis after LTBI screening

**Fig. 1:**
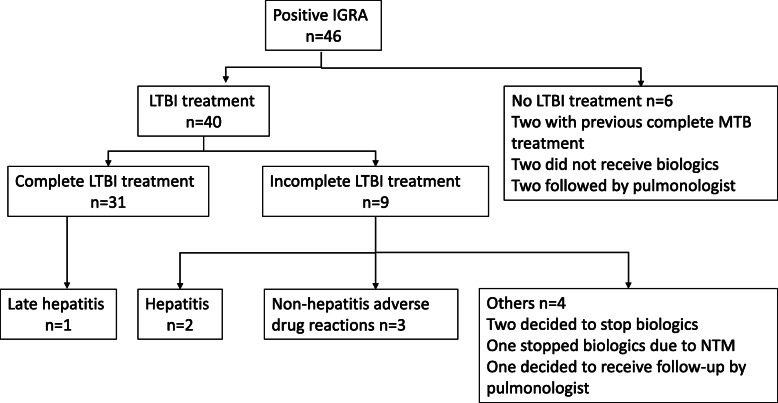
The chemoprophylaxis after LTBI screening. Forty-six patients had positive IGRA results. Forty patients received isoniazid LTBI treatment. Thirty-one (77.5%) completed LTBI therapy and one of them had late hepatitis at the end of nine-month isoniazid therapy. Five patients prematurely stopped chemoprophylaxis due to adverse drug effects. Two patients decided to stop biologics and isoniazid prophylaxis. One stopped biologics and LTBI treatment due to NTM in sputum. Another one received chest specialist follow-up instead of chemoprophylaxis. IGRA: interferon-gamma release assay; NTM: non-tuberculous mycobacteria

Taiwan recommendations for LTBI screening and prophylaxis suggests that patients with positive IGRA tests receive isoniazid prophylaxis after a comprehensive evaluation of clinical conditions and excluding active MTB. Those with negative IGRA but CXR findings of possible MTB sequalae might receive prophylactic treatment or cautious follow-up by a tuberculosis specialist or pulmonologist. Six among 46 patients with positive IGRA did not receive prophylaxis (Fig. 1), including two postponing TNFi therapy after evaluation, two followed by pulmonologists instead of isoniazid prophylaxis, and two with previous complete MTB treatment. The other 40 patients underwent isoniazid prophylaxis. IGRA-non-positive patients with old TB-suggestive CXR comprised 13.4% (32/238) of all our rheumatic patients. They were asymptomatic, and none received chemoprophylaxis.

As shown in Figs. 1, 40 patients received isoniazid LTBI treatment after screening. Thirty-one (31/40, 77.5%) finished the full course of LTBI therapy. One of the 31 patients had hepatitis at the end of isoniazid prophylaxis. In nine patients failing to complete treatment, five patients (5/40, 12.5%) prematurely stopped chemoprophylaxis due to adverse drug reaction (ADR), including two suffered from isoniazid-induced hepatitis and three had non-hepatitis ADR (severe skin itching, headache, and blurred vision). All hepatitis events were subclinical and required no admission. The patients with isoniazid-induced hepatitis had no concomitant hepatitis B or C infection. The other four patients discontinued isoniazid chemoprophylaxis, including one receiving CXR follow-up by the pulmonologist, one with NTM infection, and two stopping biologics. The number needed to harm (NNH) with isoniazid prevention therapy was 6.7 (40/6) for all adverse drug reactions and 13.3 (40/3) for hepatitis.

### Non-tuberculous mycobacteria (NTM) colonization and tuberculosis infection during follow-up

Within the year after the IGRA screening, most patients were followed monthly at the outpatient department. CXR was followed every six months or when patients had fever or cough. Among 40 patients receiving LTBI prophylaxis, no active MTB was detected, but *Mycobacterium gordonae* was isolated in the sputum of one patient 49 days after a positive IGRA test. The biologic and LTBI treatment were interrupted. One episode of pulmonary MTB was diagnosed in a patient with negative IGRA result and radiographic fibrotic lesions, which was diagnosed 60 days after TNFi, and 116 days after the negative IGRA test (Table 3).

## Discussion

Our study showed a significant clinical burden of LTBI in a tuberculosis intermediate risk area, revealing an IGRA-positive rate of 19.3% and old tuberculosis-suggestive CXR in 18.1% of our patients. Correlation between these two tests was not significant. Though IGRA-positive rate was lower in patients with psoriatic arthritis, the age and background medication prevented a direct comparison with the rates of RA or AS patients. According to Taiwan recommendations, LTBI prophylaxis was given to those who had positive IGRA after excluding active TB by CXR, even in the absence of old-TB-suggestive CXR to prevent a possible extra-pulmonary tuberculosis reactivation. Even though IGRA is more specific than TST in rheumatic patients [11], false-positive response to NTM is still possible. For example, *M. kansasii, M. marinum,* and *M. szulgai* share specific genome areas and antigens with MTB, and few other NTM species also cross-react with antigen used in IGRA [28]. *M. gordonae* was isolated in one of our patients with a possible false positive IGRA test [29], even though a concomitant LTBI could not be excluded.

Our study showed that fibrotic lesions accounted for nearly 60% (19/32) of all old-MTB-suggestive findings in patients with negative or indeterminate IGRA results, and one of them developed active MTB two months after biologic use. Fibrotic lesions, especially in the upper lobes, have been associated with a higher risk of MTB reactivation [26, 30]. However, we usually followed these patients by CXR. First, MTB sequelae in CXR are not specific, although these findings have been widely documented [31, 32]. Radiographic lesions suggestive of healed MTB lesions might have other etiologies, such as pneumoconiosis, hypersensitivity pneumonitis, or sarcoidosis. Starting a potentially hepatotoxic agent such as isoniazid according to the non-specific CXR findings is usually challenged by some physicians, especially when these patient are asymptomatic [33]. Second, active tuberculosis might be better detected by serial CXR follow-up. If a series of CXR show stable healed TB lesions, the relative risk of progression to TB is far less [6, 31]. Immediate isoniazid prophylaxis might hinder the detection of active tuberculosis and induce isoniazid resistance [34]. Even though systemic reviews did not show evidence of isoniazid chemoprophylaxis-related MTB resistance, these small-scale studies also could not exclude the possibility of an increased risk [35]. According to 2005 British Thoracic Society (BTS) guideline [23], close follow-ups without LTBI chemoprophylaxis might be acceptable in CXR-old-TB patients in real-world practices. Although pulmonologists are skilled at interpreting CXR, Cantini and colleagues reported that only two-fifths of rheumatologists ever consult a pulmonologist when screening for LTBI [19]. Therefore, cooperation between pulmonologists and rheumatologist may improve the detection of early pulmonary MTB.

Although our study did not reveal a difference between the CXR and IGRA screening results in rheumatic patients with or without prior biologic exposure, the heterogenous composition of our rheumatic patients and prior use of different biologic with different duration may biased our results. Whether the treatment will affect the screening results by CXR and IGRA may require a design of cohort study to answer this question.

LTBI prophylaxis was finished in 77.5% of our patients. NNH of isoniazid chemoprevention was 6.7 in the current study. Subclinical hepatitis was noted in 3/40 (7.5%). In addition to adverse effects, the nine-month treatment period hinders the intention and completion of chemoprophylaxis. Three months of weekly isoniazid and rifapentine (3HP) had better treatment completion rates and less hepatotoxicity than 9H in the general population [36]. A recent study of LTBI prophylaxis in patients with rheumatoid arthritis showed higher completion rates (90.5% vs 78.3%) and lower hepatitis incidence (0% vs 8.7%) of 3HP regimen compared with 9H, which indicated that 3HP might be a better regimen for LTBI prophylaxis [37]. Due to the low incidence of tuberculosis, our case number is insufficient for evaluating the efficiency of prophylaxis policy.

## Conclusion

LTBI disease burden in rheumatic patients is substantial according to the estimation of CXR and IGRA screening. Correlation between these two tests is not significant, which implies that CXR is complementary to IGRA. CXR of IGRA-positive patients should be carefully interpreted to exclude a possible active mycobacterial infection before prophylaxis. IGRA-non-positive patients with old TB-suggestive CXR are still at risk of tuberculosis reactivation or non-tuberculous mycobacteria infection, and merited cautious follow-up by rheumatologists, tuberculosis specialists, and pulmonologists.

## Data Availability

The dataset used for current study is available from the corresponding author for further study questions.

## Abbreviations

3HP: Three months of weekly isoniazid and rifapentine
9H: Nine months of daily isoniazid
ADR: Adverse drug reaction
AS: Ankylosing spondylitis
BCG: Bacillus Calmette-Guerin
BTS: British Thoracic Society
CXR: Chest roentgenography
CXR-old-TB: Radiological findings suggesting healed tuberculosis
CXR-non-TB: Chest roentgenography without pulmonary tuberculosis-suggestive lesions
HCQ: Hydroxychloroquine
IGRA: Interferon-gamma release assay
LTBI: Latent tuberculosis infection
LEF: Leflunomide
MTB: *Mycobacterium tuberculosis*
MTX: Methotrexate
NNH: Number needed to harm
NSAID: Non-steroidal anti-inflammatory drug
NTM: Non-tuberculous mycobacteria
Pre. dose: Glucocorticoid equivalent dose in prednisolone
PsA: Psoriatic arthritis
RA: Rheumatoid arthritis
SSZ: Sulfasalazine
TNF-α: Tumor necrosis factor-alpha
TNFi: Tumor necrosis factor inhibitor
TST: Tuberculin skin test
